# Large transport *J*_c_ in Cu-sheathed Sr_0.6_K_0.4_Fe_2_As_2_ superconducting tape conductors

**DOI:** 10.1038/srep11506

**Published:** 2015-06-30

**Authors:** He Lin, Chao Yao, Haitao Zhang, Xianping Zhang, Qianjun Zhang, Chiheng Dong, Dongliang Wang, Yanwei Ma

**Affiliations:** 1 Key Laboratory of Applied Superconductivity, Institute of Electrical Engineering, Chinese Academy of Sciences, PO Box 2703, Beijing 100190, China

## Abstract

Copper sheath is the first choice for manufacturing high-*T*_c_ superconducting wires and tapes because of its high electrical and thermal conductivities, low-cost and good mechanical properties. However, Cu can easily react with superconducting cores, such as BSCCO, MgB_2_ and pnictides, and therefore drastically decrease the transport *J*_c_. Here, we report the fabrication of Cu-sheathed Sr_1−x_K_x_Fe_2_As_2_ tapes with superior *J*_c_ performance using a simple hot pressing method that is capable of eliminating the lengthy high-temperature sintering. We obtained high-quality Sr_1−x_K_x_Fe_2_As_2_ tapes with processing at 800 ^o^C for 30 minutes and measured high *T*_c_ and sharp transition. By this rapid fabrication, Cu sheath does not give rise to apparent reaction layer, and only slightly diffuses into Sr-122 core. As a consequence, we achieved high transport *J*_c_ of 3.1 × 10^4^ A/cm^2^ in 10 T and 2.7 × 10^4^ A/cm^2^ in 14 T at 4.2 K. The in-field *J*_c_ performance is by far the highest reported for Cu-sheathed high-*T*_c_ conductors. More importantly, Cu-sheathed Sr-122 tapes also showed a high *J*_e_ value of 1.0 × 10^4^ A/cm^2^ in 10 T at 4.2 K, which has reached the widely accepted practical level for applications. These results demonstrate that Cu is a very promising sheath for the practical application of pnictide conductors.

The discovery of the iron-based superconductors, with a relatively high critical temperature *T*_c_, ultrahigh upper critical fields *H*_c2_ and low anisotropy *γ*, has inspired worldwide research efforts^1^,^2^,^3^,^4^,^5^. Studies on single crystals and thin films reveal that iron-pnictides have large in-field critical current density *J*_c_ due to strong intrinsic flux pinning^6^,^7^,^8^. To explore the potential of using this new material for magnet applications, significant efforts had been focused on developing wires and tapes with in-field high *J*_c_. Our research group prepared LaFeAsO_1−x_F_x_ and SmFeAsO_1−x_F_x_ superconducting wires using *in-situ* powder-in-tube (PIT) method^5^. A Ta tube or Fe tube with an inner Ti sheath was used to prevent the reaction between the tubes and the superconducting compounds. Zhang *et al.* systematically studied the effect of various sheath materials (Nb, Ta and Fe/Ti) on the microstructure and superconducting properties of SmFeAsO_1−x_F_x_ wires and reported consistent formation of a thick reaction layer between the core and metal sheath, which hinders the performance of transport *J*_c_^9^. Therefore, a key issue is to develop a technology that result in little or no reaction with iron-pnictides. Subsequently, Wang *et al.* reported Ag-sheathed Sr_1−x_K_x_Fe_2_As_2_ (Sr-122) conductors with transport *J*_c_ of 1200 A/cm^2^ (at 4.2 K and self-field)^10^. The Sr-122/Ag interface was clear and no observable reaction layer, indicating that Ag sheath is benign in proximity to the compound at high sintering temperature. Fe-sheathed Sr-122 and Ba_1−x_K_x_Fe_2_As_2_ (Ba-122) tapes were also fabricated by *ex-situ* PIT method^11^,^12^,^13^. Through optimization of sintering process and rolling texture, *J*_c_ of >10^4^ A/cm^2^ in 10 T at 4.2 K was reported for Sr-122 tapes^14^. Recently, Ag-sheathed Ba-122 and Sr-122 conductors have been investigated intensively, and high *J*_c_ exceeding 10^4^ A/cm^2^ (Maximum *J*_c_ = 1.2 × 10^5^ A/cm^2^) at 4.2 K and 10 T has been reported by applying uniaxial pressing, such as hot pressing (HP) and cold pressing (CP)^15^,^16^,^17^,^18^,^19^. By far, all pnictide wires and tapes with the high *J*_c_-*B* performance (>10^4^ A/cm^2^, at 4.2 K and 10 T) are prepared by using the expensive Ag or magnetic Fe sheath, which is similar to Ag-sheathed BSCCO and Fe-sheathed MgB_2_ conductors^20^,^21^,^22^,^23^.

For practical applications of high-*T*_c_ superconductors including cuprate, pnictide and MgB_2_, the copper material is a desirable sheath because of many advantages^24^,^25^,^26^. Firstly, when compared with common Ag and Fe sheath, Cu is a low-cost and nonmagnetic material. Secondly, Cu sheath has good mechanical properties, which make the coil winding easier in magnet applications. Thirdly, high purity Cu has large residual resistivity ratio (RRR) value, and provides both electromagnetic stabilization against flux jumps and quench protection^27^,^28^. It is well known that Cu material has been proved to be an effective sheath in the conventional NbTi and NbSn_3_ conductors. Cu is also used as stabilizer in high-*T*_c_ conductors, such as Nb/Cu/monel MgB_2_ wires^29^,^30^, for providing electrical stability of magnets and other devices during transients. However, since the discovery of cuprate superconductors in 1986, no high transport *J*_c_-*B* performance (>10^4^ A/cm^2^, at 4.2 K and 10 T) has been reported for Cu-sheathed high-*T*_c_ superconductors. Cu is highly reactive to superconducting core at high-temperature sintering^31^,^32^,^33^. The interfacial reaction layer and composition deviation of superconducting phase can lead to *J*_c_ degradation. In worse case, no transport *J*_c_ can be detected, because the thick reaction layer apparently prevented electric current from flowing from the sheath material to the superconducting core. Therefore, it is considered a grand challenge to develop a process for Cu-sheathed high-*T*_c_ superconductors with superior performance. In the present work, we report successfully fabricated Cu-sheathed Sr-122 tapes by an *ex-situ* PIT method. DC susceptibility of Sr-122 precursor powders was measured, and the result is shown in Fig. 1. The significant shielding currents appear at about 36.0 K and increase as the temperature decreased, which is similar to that reported for high-quality precursors^17^. During the final heat treatment, we introduce a hot pressing process with combination of short-time sintering (800 °C/30 min or 700 °C/60 min) and low external pressure (~20 MPa). This rapid fabrication can effectively avert the formation of reaction layer, and therefore result in a high transport *J*_c_ of 3.1 × 10^4^ A/cm^2^ at 4.2 K and 10 T.

## Results

**Figure 2:**
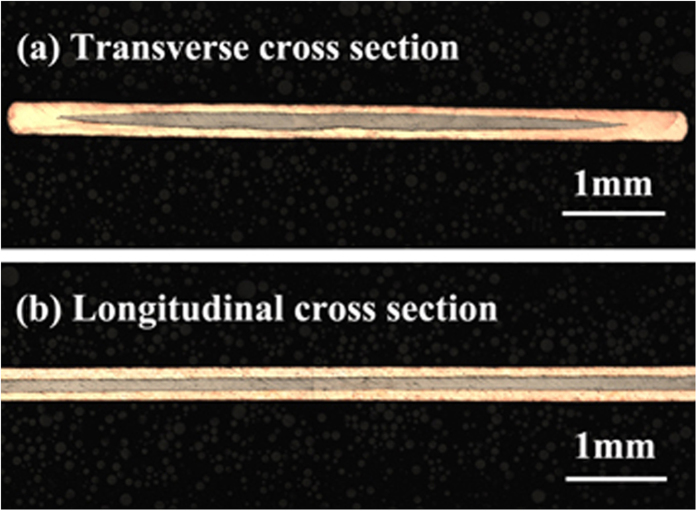
Optical images of Cu-sheathed Sr-122 tapes: (**a**) the transverse cross section of HP800 tapes; (**b**) the longitudinal cross section of HP800 tapes.

Cu-sheathed Sr-122 tapes were finally hot pressed at 700 °C (HP700 tapes) and 800 °C (HP800 tapes). Fig. 2(a) shows a typical transverse cross-sectional optical image of HP800 tapes. After hot-pressing, the tape thickness of HP800 samples decreased from ∼0.40 mm to ∼0.29 mm. Fig. 2(b) displays a longitudinal cross-sectional optical microstructure of HP800 tapes. A uniform deformation of both superconducting core and Cu sheath along the length can be obviously seen, which is essential for the achievement of high transport *J*_c_^34^,^35^. This uniformity is attributable to the good mechanical properties of Cu sheath.

**Figure 3:**
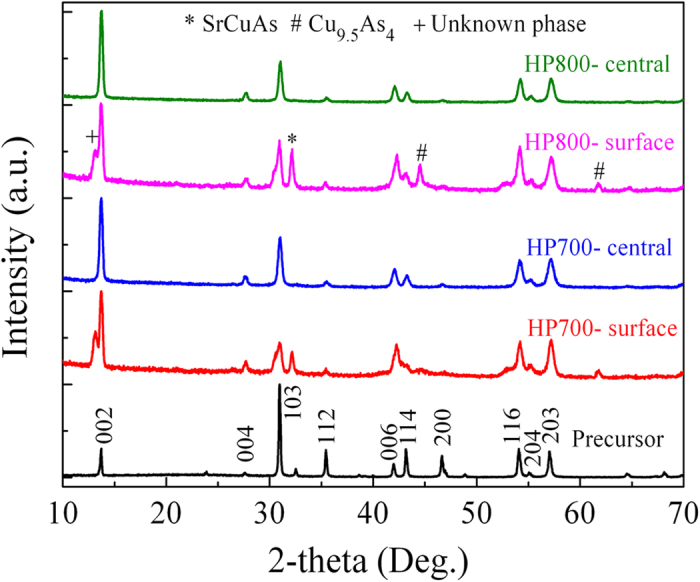
XRD patterns for the planar surfaces and central sections of the superconducting cores of both HP700 and HP800 samples. As a reference, the data for randomly orientated precursor powders is also included. The peaks of Sr_1−x_K_x_Fe_2_As_2_ phase are indexed, while the peaks of impurity phases are also marked.

As shown in Fig. 3, the XRD analysis was performed on the planar surfaces of superconducting cores after peeling off Cu sheath. For comparison, the data for randomly orientated precursor powders is also included. The XRD patterns on the surfaces clearly exhibit a ThCr_2_Si_2_-type structure, ensuring that Sr-122 is the main phase for both HP700 and HP800 samples. Using a final short-time hot-pressing process, the formation of non-superconducting reaction layer at the interface seems to be prevented. More importantly, the transport critical current *I*_c_ may be measured and obtained in these Sr-122 tapes^31^. However, the impurity peaks are detected on the core surface, especially for HP800 samples. Some Cu reacted with Sr-122 phase, producing SrCuAs and Cu_9.5_As_4_ phases. This is consistent with large FWHM (full width at half-maximum) of (002) and (103) peaks for Sr-122 phase. On the other hand, the XRD patterns for the central planar sections of HP tapes after carefully polishing are also exhibited in Fig. 3. The diffraction peaks have some differences compared to those of the surfaces. The XRD patterns of central parts exhibit pure Sr-122 phase without detectable impurities. No Cu element can be detected in the central parts by further EDX identification. The peak characteristics are similar to those of textured Sr-122 tapes^17^,^18^,^19^, which have high transport *J*_c_-*B* properties. We quantify the c-axis texture parameter *F* according to the Lotgering method^36^ with *F* = (ρ – ρ_0_)/(1 – ρ_0_), where ρ = ∑*I*(00*l*)/ *I*(hk*l*) and ρ_0_ = ∑*I*_*0*_(00*l*)/ *I*_*0*_(hk*l*). *I* and *I*_0_ are the intensities of corresponding XRD peaks measured for the textured and randomly oriented samples, respectively. *F* values of 0.41 and 0.44 were obtained for HP700 and HP800 tapes, demonstrating that c*-*axis oriented grains have been achieved in Cu-sheathed tapes. The larger *F* value in HP800 samples is in agreement with the previous reports confirming that the higher HP temperature, the larger degree of grain alignment^18^.

**Figure 4:**
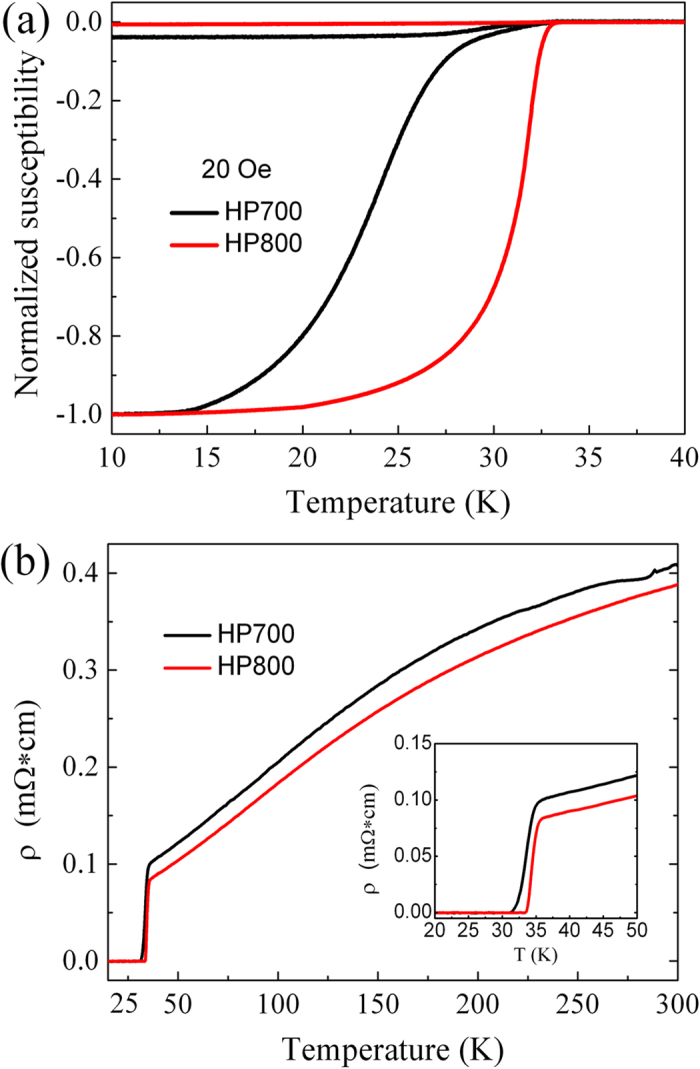
(**a**) Temperature dependence of the DC magnetic susceptibility curves of HP700 and HP800 samples. (**b**) Resistivity versus temperature curves of HP700 and HP800 samples; Inset showing the enlarged part near the superconducting transition. All data were obtained after peeling off the Cu sheath.

DC susceptibility measurements were conducted on HP700 and HP800 samples. Fig. 4(a) depicts two typical groups of the susceptibility curves under a 20 Oe magnetic field parallel to the tape plane. The superconducting transition of HP700 tapes begins at about 33.0 K. It is evident from the zero-field cooled (ZFC) signal that the susceptibility starts to decrease slowly and full shielding is reached at about 15 K. This behavior suggests the presence of inhomogeneity^14^,^37^. For HP800 samples, the shielding current occurs at 33.5 K, which may be ascribed to improvement in crystallization. When compared to HP700 samples, the HP800 samples exhibit sharper superconducting transition and reach full shielding at higher temperature (≈20 K). Obviously, enhanced uniformity in superconducting phase has been achieved in HP800 samples^37^. Fig. 4(b) shows resistivity versus temperature curves. We measured onset *T*_c_ values of 34.6 and 35.1 K for HP700 and HP800 tapes, respectively, which are comparable to Fe-sheathed and Ag-sheathed tapes^17^,^19^,^38^, but slightly smaller than those reported in ref. 18. The impurity of copper compound in present work does not significantly affect the superconducting transition. In addition, the resistivity of HP700 and HP800 tapes drops to zero *T*_*c*_ at 32.3 and 33.8 K, respectively. The larger onset *T*_c_ and smaller transition width for HP800 samples are consistent with the above magnetic results.

**Figure 5:**
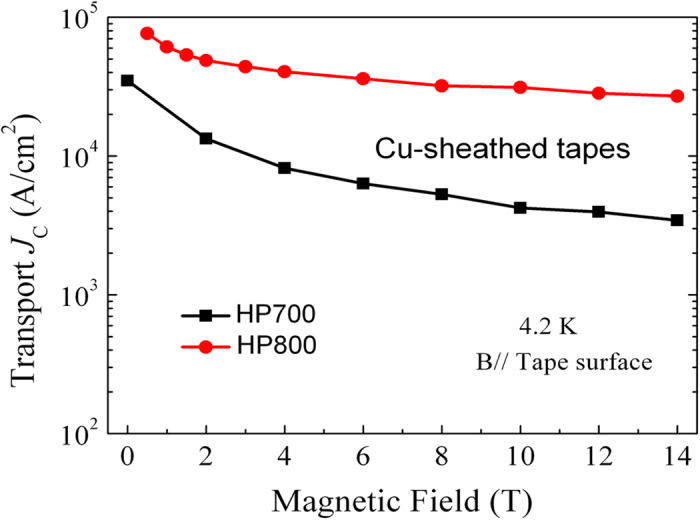
Magnetic field dependence of the transport *J*_c_ at 4.2 K for both HP700 and HP800 tapes. The applied fields up to 14 T were parallel to the tape surface.

From the viewpoint of practical applications, superconducting wires must be able to carry large transport current density in high magnetic fields. We determined the transport *I*_c_ by the standard four-probe method. Fig. 5 displays the *J*_c_-*B* properties of HP700 and HP800 tapes at 4.2 K. For HP700 tapes, the *J*_*c*_ values of 3.5 × 10^4^ A/cm^2^ and 4.2 × 10^3^ A/cm^2^ are obtained in self-field and 10 T, respectively. The striking result is that HP800 tapes show a great enhancement of *J*_*c*_ values in the whole field up to 14 T. Such an improvement can be attributed to improved texture, better homogeneity and crystallization. For HP800 tapes, the *J*_c_ data in self-field is not given because the transport *I*_c_ is too large to be measured by the measurement system we used. Excitingly, the transport *J*_c_ reaches 3.1 × 10^4^ A/cm^2^ at 10 T. To our knowledge, this is by far the highest critical current density under high field ever reported for Cu-sheathed high-*T*_c_ superconductors. Importantly, due to its extremely small magnetic field dependence, the transport *J*_*c*_ still maintains a high value of 2.7 × 10^4^ A/cm^2^ in 14 T. It is convincible that the Cu-sheathed Sr-122 tapes have a very promising future for use in high-field superconducting magnets.

**Figure 6:**
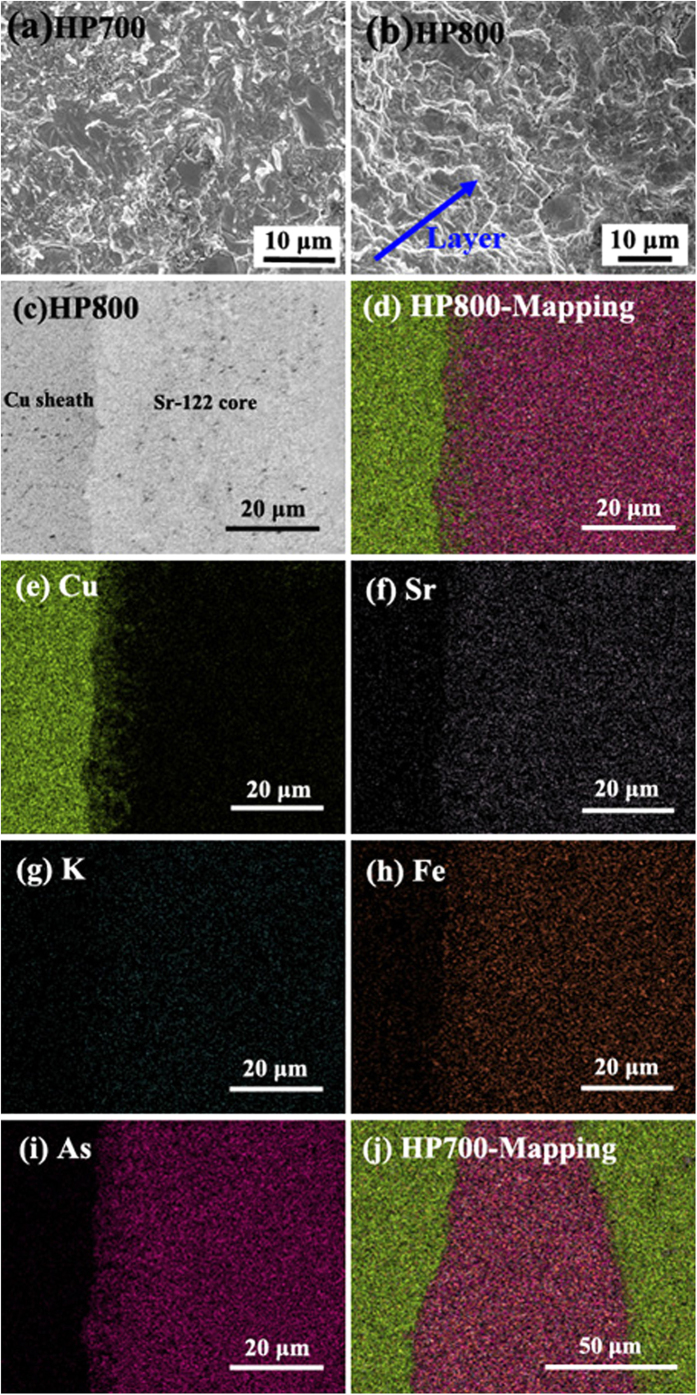
SEM microstructures of Sr-122 tapes: (**a**) HP700 and (**b**) HP800 samples. (**c**) SEM image showing the interface between Cu sheath and Sr-122 core for HP800 tapes. (**d**) The corresponding EDX mapping image for HP800 samples; (**e–i**) Area mappings of Cu, Sr, K, Fe and As element, respectively. (**j**) The EDX mapping image for HP700 samples.

We conducted SEM/EDX to investigate the influence of hot pressing process on the microstructure of Cu-sheathed Sr-122 tapes. As shown in Figs. 6(a,b), both HP700 and HP800 samples exhibit dense layered structure, which is similar to that of Bi-2223 tapes. HP700 samples have smaller grain size than that of HP800 samples. Fig. 6c exhibits a typical SEM micrograph of polished cross section of HP800 samples. It is noted that the boundary between Cu sheath and Sr-122 core is clear, further suggesting that there is no apparent reaction layer after hot pressing^13^. The corresponding EDX element mappings of HP800 tapes are presented in Figs. 6(d–i). From the Cu mapping, we observe a diffusion of Cu into Sr-122 area, and the diffusion width is approximately 8 μm. This indicates that Cu element interfuses into Sr-122 core and reacts with it during heat treatment. For the elements of Sr-122 phase, Sr, K, Fe and As are detected locally in the core area, disappear almost completely at the border of the core area. Comparing with recent Ag-sheathed Sr-122 tapes^18^, we conclude that the slight depression of superconducting properties in this work is mainly due to the diffusion of Cu. At the same time, the diffusion also causes the inhomogeneous distribution of the superconducting elements in Sr-122 area, particularly in the diffusion region. In addition, the EDX mapping of HP700 samples is showed in Fig. 6(j). For each element, the content has a dramatic change at the border of Sr-122 core. Further analysis reveals that the diffusion width of Cu element is smaller than 3 μm in HP700 samples. Although the sintering time of 60 min is longer than that used for HP800 tapes (30 min), the width is much smaller.

The diffusion of Cu and the composition deviation of superconducting phase easily induce severe porosity at the interface, and apparently break the electrical contact between Cu sheath and Sr-122 core^39^,^40^. This disadvantageous phenomenon can be avoided by the simple HP method, because it can greatly reduce the pores and cracks by combining the deformation and heat treatment in a single step. As shown in Fig. 6c, the Cu sheath and Sr-122 core are tightly connected. As a result, high transport *I*_c_ values have been measured in our Cu-sheathed tapes.

For comparison, Cu-sheathed Sr-122 tapes were also sintered without hot-pressing, and the detailed information is exhibited in Table 1. The transport *J*_c_ values for both HP tapes are much larger than those of corresponding tapes without hot-pressing. For example, the *J*_c_ value of HP800 tapes (3.1 × 10^4^ A/cm^2^) is an order of magnitude higher than that of R800 tapes (3.0 × 10^3^ A/cm^2^), indicating the great *J*_c_ enhancement by the hot-pressing method.

## Discussion

Using copper sheath for superconducting tapes with large transport *J*_c_ is highly desirable for practical applications. By a modified hot-pressing method with combination of final short-time sintering and low external pressure, we successfully prepared Cu-sheathed Sr-122 conductors with large transport current. We demonstrated that the fabricating method developed in our lab can produce high-performance Cu-sheathed superconductors. First, a short-time hot-pressing process can form high-quality Sr-122 phase, which is supported by XRD and resistivity characterizations. For HP800 tapes, the resistivity data demonstrates that the onset *T*_c_ is 35.1 K with a transition width of about 1.5 K. Second, this fast fabrication does not give rise to the reaction layer even though the Cu sheath is used. As discussed by above EDX mappings, only a little bit of Cu diffuses into polycrystalline Sr-122 phase. Earlier studies reveal that the thick reaction layer induces the contamination of the superconducting phase to decrease *T*_c_, and prevents electric current from flowing from the sheath material to the superconducting core^26^,^33^. Third, the Cu sheath and Sr-122 core are tightly connected under external pressure, and thus the current path can be enlarged. Meanwhile, the hot pressure can not only considerably increase the core density, but also effectively promote complete reaction of Sr-122 phase, which in return to solve the problem that is low sintering temperature (800 or 700 °C) and short-time reaction (30 or 60 min) yield poor re-crystallization and ordinary superconducting performance^18^,^38^. In summary, the simple hot pressing method ensures high-quality Sr-122 phase and inhibit the formation of reaction layer in Cu-sheathed Sr-122 tapes.

**Figure 7:**
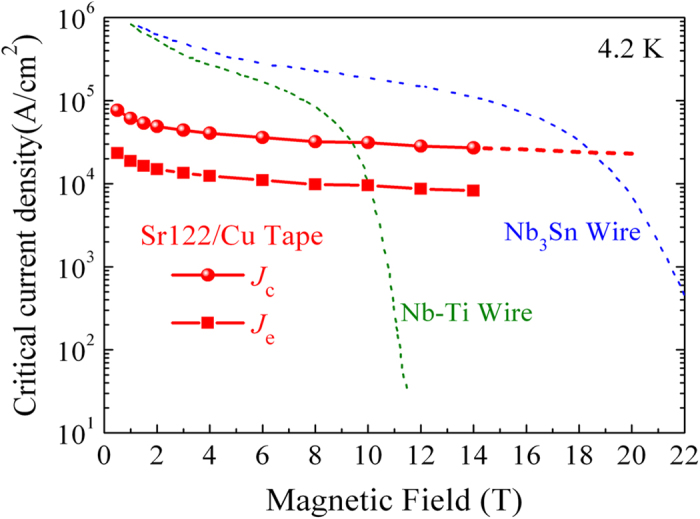
Magnetic field dependence of the transport critical current density *J*_c_ and the engineering current density *J*_e_ for our best Sr122/Cu tapes. The *J*_c_-*B* curves of PIT processed NbTi and Nb_3_Sn wires are also shown for comparison.

It is fascinating that the largest *J*_c_ value of 3.1 × 10^4^ A/cm^2^ in 10 T has been obtained in our best Cu-sheathed tapes. Moreover, the *J*_c_ of 122-type pnictides have very weak field dependence in strong fields up to 28 T^41^, in accordance with ultrahigh *H*_c2_ values^5^. Thus, the *J*_c_ data above 14 T is given by extrapolating from low fields, as presented in Fig. 7. The curve tendency shows that the crossovers with Cu-sheathed NbTi and Nb_3_Sn wires are around 9.5 and 18.5 T, respectively. This clearly strengthens the position of pnictide conductors as a competitor to the conventional superconductors for high-field applications. On the other hand, researchers are usually more concerned with the engineering (total cross section) current density *J*_e_ in practical applications. As showed in Fig. 7, a high *J*_e_ of about 1.0 × 10^4^ A/cm^2^ at 10 T has been achieved in our Cu-sheathed Sr-122 tapes, which has reached the widely accepted practical level for applications^28^. This achievement is a significant technical breakthrough for the practical applications of Cu-sheathed high-*T*_c_ conductors. In the future, if the HP process can be properly adjusted to match the balance between the well re-crystalline reaction and little impurity phase, an even higher *J*_e_ can be expected.

The specific cost ($/kA·m) of superconducting wires and tapes must be considered in practical applications^25^,^28^. The price ($/kg) of Cu metal is 1-2 order of magnitude lower than that of expensive Ag metal. Tape conductors with high *J*_e_ (10^4^ A/cm^2^) sheathed in comparatively cheap copper have the strong potential for low specific cost. Moreover, Cu-sheathed conductors do not need additional stabilization or mechanical reinforcement. In contrast, the Ag sheathed wires usually need mechanical reinforcement. For example, the stainless steel or Ag0.5 wt%Al alloy sheath have been used in superconducting wires^41^,^42^,^43^, which decrease the engineering *J*_e_ or increase the complexity and cost of fabrication process. From these view points, we can conclude that the comprehensive performances of our Cu-sheathed Sr-122 tapes are much more attractive for applications than the reported Ag sheathed tapes^16^,^17^,^18^,^19^, demonstrating that Cu is a very promising sheath for the pnictide wires and tapes.

## Methods

### Sample preparation

We fabricated Cu-sheathed Sr_0.6_K_0.4_Fe_2_As_2_ tapes by *ex-situ* PIT method. Sr fillings, K pieces, and Fe and As powder with a ratio of Sr:K:Fe:As = 0.6: 0.5: 2: 2.05 were mixed for 12 hours by ball-milling method. The milled powders were packed into Nb tubes and then sintered at 900 °C for 35 h. As prepared Sr-122 superconducting powders were packed into Cu tubes with OD 6 mm and ID 4 mm. These tubes were sealed and then cold worked into tapes (~0.4 mm thickness) by swaging, drawing and flat rolling. Finally, hot pressing was performed on the 60 mm long tapes under ~20 MPa at two different sintering processes of 800 °C/30 min and 700 °C/60 min. These tapes are defined as HP800 and HP700 tapes, respectively.

### Measurements

Phase identification of samples was characterized by X-ray diffraction (XRD) analysis with Cu Kα radiation. Magnetization versus temperature curves and resistivity measurements of the superconducting cores were carried out using a PPMS system. The cross sections were polished and then observed by optical images. Microstructure characterization was analyzed using SEM images and EDX scanning. The transport critical current *I*_c_ was measured at 4.2 K using short tape samples of 3 cm in length with the standard four-probe method and evaluated by the criterion of 1 μV/cm. The applied fields up to 14 T in transport *I*_c_ measurement were parallel to the tape surface.

## Additional Information

**How to cite this article**: Lin, H. *et al.* Large transport *J*_c_ in Cu-sheathed Sr_0.6_K_0.4_Fe_2_As_2_ superconducting tape conductors. *Sci. Rep.* **5**, 11506; doi: 10.1038/srep11506 (2015).

## Figures and Tables

**Figure 1:**
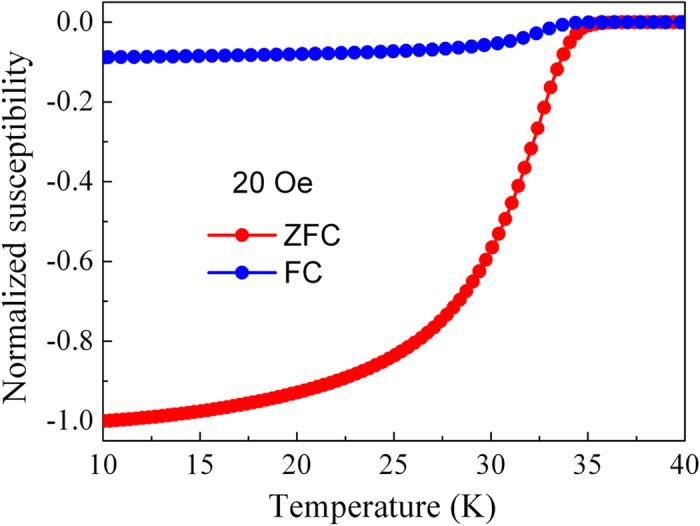
Magnetization versus temperature curves of the precursor powders.

**Table 1:** The onset *T*_c_ and transport *J*_c_ for Cu-sheathed Sr-122 tapes with and without hot pressing.

Sample name	Fabrication conditions	Onset *T*_c_ (K)	*J*_c_ (kA/cm^2^) (4.2 K, 10 T)
R700	700 °C/1 h	34.3	1.3
R800	800 °C/0.5 h	34.5	3.0
HP700	700 °C/1 h/20 MPa	34.6	4.2
HP800	800 °C/0.5 h/20 MPa	35.1	31.0
